# Regulation of Apoptosis During Porcine Circovirus Type 2 Infection

**DOI:** 10.3389/fmicb.2018.02086

**Published:** 2018-09-04

**Authors:** Yuhong Pan, Pengfei Li, Renyong Jia, Mingshu Wang, Zhongqiong Yin, Anchun Cheng

**Affiliations:** ^1^Research Center of Avian Disease, College of Veterinary Medicine, Sichuan Agricultural University, Chengdu, China; ^2^Institute of Preventive Veterinary Medicine, Sichuan Agricultural University, Chengdu, China; ^3^Key Laboratory of Animal Disease and Human Health of Sichuan Province, Chengdu, China

**Keywords:** apoptosis, PCV2, viral proteins, signaling pathway, mechanisms

## Abstract

Apoptosis, an indispensable innate immune mechanism, regulates cellular homeostasis by removing unnecessary or damaged cells. It contains three signaling pathways: the mitochondria-mediated pathway, the death receptor pathway and the endoplasmic reticulum pathway. The importance of apoptosis in host defenses is stressed by the observation that multiple viruses have evolved various strategies to inhibit apoptosis, thereby blunting the host immune responses and promoting viral propagation. Porcine Circovirus type 2 (PCV2) utilizes various strategies to induce or inhibit programmed cell death. In this article, we review the latest research progress of the apoptosis mechanisms during infection with PCV2, including several proteins of PCV2 regulate apoptosis via interacting with host proteins and multiple signaling pathways involved in PCV2-induced apoptosis, which provides scientific basis for the pathogenesis and prevention of PCV2.

## Introduction

Porcine circovirus (PCV) from the genus *Circovirus* within the family *Circoviridae* is an icosahedral, small, non-enveloped DNA virus with a circular, single negative-stranded genome of approximately 1.76 kb (Tischer et al., 1982; Fauquet et al., 1995; Wei et al., 2016; Wang et al., 2018). To date, three species of PCV have been confirmed: Porcine circovirus type 1 (PCV1), PCV2 and Porcine circovirus type 3 (PCV3) (Alarcon et al., 2013; Segalés et al., 2013). PCV1 was first discovered in 1974 and widely acknowledged to be non-pathogenic (Tischer et al., 1982). while PCV2 was the causative agent of PCVAD/PCVD, which include reproductive failure, porcine dermatitis and nephropathy syndrome, proliferative and necrotizing pneumonia and PCV2 systemic disease (PCV2-SD) (Allan et al., 1998, 1999; Ellis et al., 1998; Meehan et al., 1998; Opriessnig et al., 2007). The main immunopathological features of PCV2-SD are peripheral blood lymphopenia and T- and B-lymphocyte depletion in lymphoid tissue (Nielsen et al., 2003; Resendes et al., 2004; Resendes and Segalés, 2015; Richmond et al., 2015). What’s more, severely PCV2-infected pigs could damage immune system and trigger immunosuppression by replicating and inducing apoptosis in lymphocytes (Kiupel et al., 2005; Li et al., 2013; Bin et al., 2015), leading to poor immune response to vaccines and increased susceptibility to other infectious diseases. Hence, even though PCVAD is effectively controlled by commercial vaccines, vaccination does not eliminate infection (Fort et al., 2008; Opriessnig et al., 2008, 2010). PCV2 is also one of the most important viruses in all pig-raising areas and is increasingly considered as a serious threat to global pig industry (Segalés et al., 2005; Xiang-Jin, 2013; Salgado et al., 2014; Zhai et al., 2014; Xiao et al., 2015; Mao et al., 2017; Liu et al., 2018). Phan et al. (2016) first isolated PCV3 from piglets with clinical disease of weight loss, swollen joints and anorexia. In addition, the dermatitis and nephropathy syndrome has been recently associated to PCV3 (however, this is still under discussion).

Porcine circovirus has 11 potential ORFs, so far to date, four of them have been characterized as functional proteins in replicating PCV2, including ORF1 to ORF4 (Hamel et al., 1998; Lv et al., 2014a; Hong et al., 2015), while only three ORFs have been recognized for PCV1 and PCV3: ORF1 to ORF3 (Saraiva et al., 2018). The ORF1 encodes two replicases (Rep and Rep’), the Rep proteins of PCV-1 and PCV-2 are similar in size and are responsible for the replication of the circoviral genome (Mankertz, 2012). The capsid protein encoded by ORF2 is the sole structural protein of PCV2 and contains a highly conserved basic amino acid sequence (Timmusk et al., 2008; Latini et al., 2011); therefore, it contains the major antigenic determinants of the virus (Nawagitgul et al., 2000). However, the three proteins of PCV3 are less similar to those of PCV1 and PCV2 (Palinski et al., 2016; Phan et al., 2016). The proteins encoded by ORF3 and ORF4 genes are not required for viral replication, but are closely related to the spread and virulence of the virus (Lv et al., 2015a). The protein encoded by ORF3 gene plays a vital role in the pathogenesis of the virus through its apoptotic activity *in vitro* and *in vivo* (Liu et al., 2005, 2006; Lin et al., 2011). The ORF4 protein is capable of blocking PCV2-induced apoptosis by bringing down caspases activities (Gao et al., 2014a; Lv et al., 2015b). Besides this, a novel ORF5 protein has recently been discovered in PCV2-infected cells and may be involve in activation of NF-κB pathway (Lv et al., 2015a).

Apoptosis, also called programmed cell death, is an indispensable defense mechanism for host resistance to pathogens invasion (Jorgensen et al., 2017). Apoptosis is strictly regulated and can be triggered by multiple stimuli such as normal development, pathogen infection and several factors leading to disruption of cellular functions (Tait and Green, 2010; Czabotar et al., 2014). Apoptotic cells exhibit characteristic morphological abnormalities including chromatin condensation, nuclear fragmentation, membrane blebbing, and apoptotic body formation (Kroemer et al., 2005; Galluzzi et al., 2007). Apoptosis classically occurs via the intrinsic pathway (also called the mitochondrial pathway), the extrinsic pathway (also called the death receptor pathway) and the ER pathway (Hong et al., 2015). In brief, the mitochondrial pathway is induced by a variety of intracellular signals, such as hypoxia, nutrient deprivation and oxidative stress, which cause MOMP (Kroemer et al., 2006). Subsequently, AIF, cyt c and Smac/DIABLO are released from the mitochondrial membrane to the cytoplasm (Kroemer et al., 2006; Galluzzi et al., 2012). Cyt c can recruit pro-caspase9 and apoptotic protease activating factor-1 (apaf-1) to form an apoptosome, which subsequently activates downstream executioner caspases to trigger apoptosis (Li et al., 1997; Acehan et al., 2002). In addition, the mitochondrial pathway is mainly regulated by Bcl-2 (B-cell lymphoma 2) family proteins, which are classified into three types (Cory and Adams, 2002). One is an anti-apoptotic sub-family, which includes Bcl-xL (B-cell lymphoma-extra large) and Bcl-2. Another is pro-apoptotic BH3 only proteins, such as Bid (BH3 interacting-domain death agonist) and Bad (Bcl-2 associated death promoter), these proteins are antagonists to the anti-apoptotic sub-family proteins. The third sub-family includes Bak (Bcl-2 homologous antagonist killer) and Bax (Bcl-2 associated x protein). On the other hand, the death receptor pathway is activated by the binding of a specific ligand to the corresponding death receptor, resulting in activation of caspase8 and caspase3, which finally leads to cleavage of cellular DNA (Lamkanfi et al., 2007; Tummers and Green, 2017). What’s more, ER stress regulates the concentration of Ca^2+^ and initiates the IRE1, PERK, and ATF6 pathways, which are associated with the mitochondrial pathway of apoptosis (Shore et al., 2011; Verma and Datta, 2012).

Porcine Circovirus type 2 infection induces apoptosis both *in vitro* and *in vivo* (Chang et al., 2007a; Seeliger et al., 2007; Galindocardiel et al., 2011; Resendes et al., 2011; Sinha et al., 2012), it has been reported that PCV2 can induce B lymphocyte deletion through apoptosis and macrophage apoptosis can be detected in the spleen of PCV2 infected mice (Shibahara et al., 2000), so apoptotic cell death may be one of the causes of lymphopenia after PCV2 infection (Resendes et al., 2004). Similarly, apoptosis is one of the causes of lymph node loss and hepatocyte decline in pigs with PMWS (Krakowka et al., 2004). Apoptosis has also been proposed as a natural part of the viral life cycle (Young et al., 2007), in the early stage of PCV2 infection, PCV2 may prevent apoptosis by expressing its anti-apoptotic gene to accomplish its propagation, while apoptosis may be a powerful strategy for the release and dissemination of progeny virions in the late stage (Liu et al., 2005). However, the molecular mechanism of PCV2-regulated apoptosis is still unclear. In this article, we focus on reviewing the roles of PCV2 in the process of apoptosis, which is useful for future research.

## Viral Proteins and Their Apoptosis Regulation Mechanisms

### Cap and Its Mechanism of Apoptosis Regulation

#### The Interactions Between Cap and Cellular Proteins

The ORF2 36 gene encodes the major immunogenic capsid protein of 27.8 kDa. By investigating the replication and pathogenesis mechanisms of PCV2, the interactions between the PCV2 Cap protein with nine different cellular proteins were confirmed (**Table 1**), including complement factor C1qB, the receptor protein for the gC1qR, MKRN1, cell adhesion molecule P-selectin, prostate apoptosis response-4 (Par-4) protein, NAP1, NPM1, Hsp70 and Hsp40 (Timmusk et al., 2006a; Finsterbusch et al., 2009b; Liu et al., 2013b). However, only MKRN1 and Hsp70 have been confirmed to participate in PCV2-induced apoptosis.

**Table 1:** Interactions of cellular proteins with the proteins of PCV2 (Lv et al., 2014).

PCV2 protein	Cellular interacting	Functions	Reference
	**proteins**		
ORF1/Rep protein	Syncoilin	Transport processes	Timmusk et al., 2006a
	c-myc	Transcriptional regulation	Timmusk et al., 2006a
	ZNF265	Altemative splicing	Finsterbusch et al., 2009a
	TDG	transcriptional regulation, DNA repair	Finsterbusch et al., 2009a
	VG5Q	Angiogenesis	Finsterbusch et al., 2009a
ORF2/Cap protein	C1qB	Complement factor	Timmusk et al., 2006a
	P-selectin	Cell adhesion molecule	Timmusk et al., 2006a
	gC1qR	C1qB receptor, multifunctional	Finsterbusch et al., 2009a; Du et al., 2016; Kouokam Fotso et al., 2016
	MKRN1	E3 ubiquitin ligase	Finsterbusch et al., 2009a
	NAP1	Transport, chaperonin	Finsterbusch et al., 2009a
	Par-4	Apoptosis, transport, cell mobility	Finsterbusch et al., 2009a
	NPM1	Ribosome biogenesis	Finsterbusch et al., 2009a
	Hsp40	Chaperonin	Finsterbusch et al., 2009a
	Hsp70	Chaperonin	Liu et al., 2013b
ORF3 protein	DDE-like transposase	Transposase	Timmusk et al., 2006a
	poRGS16	Cell signaling, nuclear transport of ORF3	Levine and Oren, 2009 ; Hsu et al., 2010
	pPirh2	E3 ubiquitin ligase	Liu et al., 2007
ORF4 protein	FHC	Ferroxidase	Lv et al., 2015b, 2016
	SNRPN	Pre-Mrna splicing	Xiao et al., 2015
	COX8A	COX subunit	Xiao et al., 2015
	Lamin C	Intermediate filament protein	Xiao et al., 2015; Lin et al., 2018
	ANT3	Adenine nucleotide translocase	
ORF5 protein	GPNMB	Transmembrane glycoprotein	Lv et al., 2015a
	CYP1A1	Cytochrome P450 enzyme	Lv et al., 2015a
	YWHAB	Adapter protein	Lv et al., 2015a
	ZNF511	Transcriptional regulator	Lv et al., 2015a
	SRSF3	RNA splicing factor	Lv et al., 2015a

#### Apoptosis Regulated by Cap and MKRN1

According to Gray et al. (2000) and Lee et al. (2013), MKRN1 is a transcriptional co-regulator and an E3 ubiquitin ligase that is highly evolutionarily conserved in vertebrates, it can also mediate apoptosis and p53-dependent cell cycle arrest. The interactions between different types of PCVs and their hosts have been analyzed by Finsterbusch’s group, the research demonstrated that MKRN1 can interact with Cap proteins of both PCV1 and PCV2, resulting a decreased concentration of MKRN1 in the host (Finsterbusch et al., 2009a). The decreased MKRN1 can in turn reduce the level of p53 ubiquitination, resulting in an increase of p53 and thus promote cellular apoptosis. Under normal conditions, p53 and p21 both are suppressed by MKRN1 through ubiquitin-dependent degradation (Lee et al., 2009). Previous studies have showed that p21 is capable of activating cell cycle arrest via suppressing apoptosis (Gartel and Tyner, 2002; Javelaud and Besancon, 2002; Abbas and Dutta, 2009; Jung et al., 2010). Therefore, ubiquitination and degradation of p21 mediated by MKRN1 may also contribute to trigger apoptosis. However, under stresses such as DNA damage, only p53 is suppressed by MKRN1; p21, which is not ubiquitinated by MKRN1 can also inhibit p53. Therefore, when the suppression of p53 by MKRN1 and p21 is reduced, the concentration of p53 will increase and thus promotes the apoptotic process (Lee et al., 2009).

#### Apoptosis Regulated by Cap and Hsp70

**FIGURE 1:**
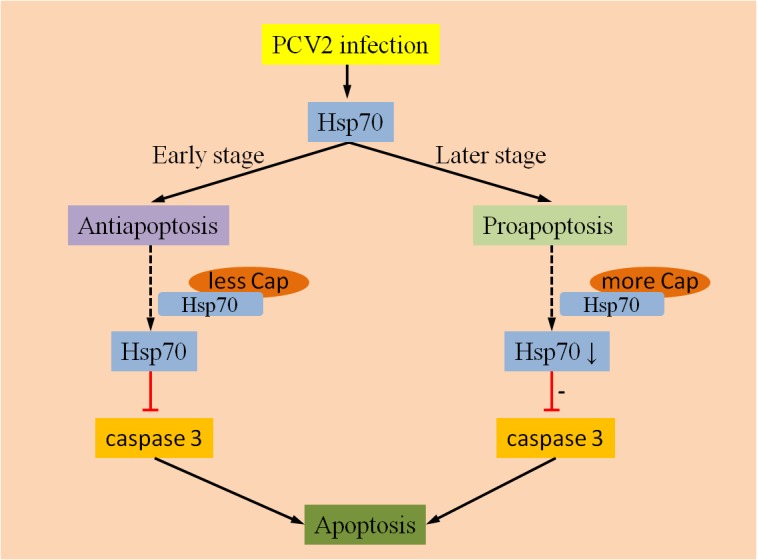
Anti-apoptotic and pro-apoptotic activities of Hsp70. Hsp70 can be activated by PCV2 infection, whereas Hsp70 mainly exert an anti-apoptotic function at an earlier stage of infection due to few interactions with PCV2 Cap proteins. During later stages of infection, anti-apoptotic responses might be weakened by down-regulation of Hsp70 due to the increased interaction with the PCV2 Cap protein.

Hsp70 is a chaperone whose expression is induced by a variety of stimuli. A previous study confirmed that Hsp70 could inhibit the production of apoptosome and apoptosis in varying degrees by suppressing the activity of AIF (Garrido et al., 2006). In study of PMWS pathogenesis using proteomics strategies, Ramírez-Boo et al. (2011) reported that the down-regulation of Hsp70 was detected in inguinal lymph nodes of piglets after inoculation with PCV2. Another study regarding the interaction between PCV2 and target immune cells showed the expression of Hsp70 was up-regulated in PAMs during the initial stage of PCV2 infection (Liu et al., 2013a). A recent study showed that the PCV2 Cap protein can interact with Hsp70 and activate it further, then apoptosis could be inhibited via blockage the activation of caspase-3 in 3D4/31 cells (Liu et al., 2013b). However, the interaction between the PCV2 Cap protein and Hsp70 may reduce Hsp70 levels, and activate caspase-3 which plays a key role in the execution of apoptosis. Accordingly, Hsp70 might play different roles on different stages of PCV2 infection (**Figure 1**). For example, Hsp70 can be induced by PCV2 infection, but lack of sufficient Cap protein in the early stage of viral infection could result in inhibiting apoptosis by Hsp70. In contrast, during the later stages of PCV2 infection, the anti-apoptotic responses may be weakened by the down-regulation of Hsp70 levels due to the increased interaction with the PCV2 Cap protein.

### ORF3 and Its Mechanisms of Apoptosis Regulation

Although the PCV2 non-structural protein ORF3 is not critical for viral replication Lin et al. (2013), found that the nuclear localization of ORF3 is correlated with triggering apoptotic response in porcine PBMC, it was also involved in PCV2-induced the extrinsic apoptosis pathway through activation of the caspase8 and caspase3 (Kiupel et al., 2005; Liu et al., 2005).

During a study of modulation of cellular functions by the PCV2 ORF3 protein, the ORF3 protein was found to directly interacted with pPirh2 (also called RCHY1), Pirh2 is an E3 ubiquitin ligase targeting p53 and leading p53 to degradation. The interaction between the pPirh2 and ORF3 protein could suppress pPirh2 stabilization and increase p53 cellular levels, thereby leading to apoptosis (Leng et al., 2003; Liu et al., 2007). Furthermore, present research suggests the amino acid residues of ORF3 protein are indispensable to compete with the interaction with pPirh2 over p53 (Timmusk et al., 2008; Karuppannan et al., 2010). p53 is a tumor suppressor as well as a transcription factor (Levine and Oren, 2009); it is also involved in regulation of diverse biological responses such as apoptosis, DNA damage, cell cycle arrest, oncogenic activities, erosion of telomeres, hypoxia and other physiological processes (Vousden and Prives, 2009; Collavin et al., 2010; Chang et al., 2013). It was reported that p53 was involved in apoptosis through transcription-dependent or -independent mechanisms during stress (Li et al., 2011; Xu et al., 2016). In general, the p53 protein content in cells is maintained at a very low level in the absence of stress through binding to proteins such as MDM2 (denoted HDM2 in humans), COP1, pPirh2 and JNK, which facilitates the degradation of p53 by the ubiquitin/proteasome pathway (**Table 2**). In stress situations such as cell cycle arrest, apoptosis may be caused by a complex formed by p53 with pro-apoptotic and anti-apoptotic members of the Bcl-2 family (such as Bcl-2, Bcl-xL, Bak and Bax). Then, the complex triggers MOMP, liberating essential apoptotic factors (such as Cyt c, AIF, and Apaf-1) and ultimately causing a caspase cascade and apoptosis via the intrinsic pathway (Marchenko et al., 2007; Wolff et al., 2008; Green and Kroemer, 2009; Collavin et al., 2010).

**Table 2:** Selected interactions of cellular proteins with p53.

	Protein	Cell lines	Reference
Kinases	PLK1	COS-7, H1299	Ando et al., 2004
	JNK1/2	A549	Oleinik et al., 2007
	GSK3-beta	H1299	Watcharasit et al., 2003
	HIPK2	H1299, HeLa	Li et al., 2007
	CK1	MEFs	Alsheich-Bartok et al., 2008
	SKG2, PAK3	HFKs	Baldwin and Munger, 2010
Ubiquitin ligases	MDM2, MDMX	MEFs, H1299	Brignone et al., 2004; Wade et al., 2010; Mancini et al., 2014
	COP1	U2OS	Dornan et al., 2004
	Pirh2	MEFs, Saos2	Leng et al., 2003
	Synoviolin	HEK293T	Yamasaki et al., 2014
	CHIP	MCF-7	Li et al., 2011a
	TRIM24	U2OS, HEK293T, MCF-7	Allton et al., 2009
	E4F1	U2OS	Le Cam et al., 2006
Acetyltransferases	P300	HCT116, Saos2, H1299	Mantovani et al., 2007
	PCAF		Liu et al., 1999
	TIP60	U2OS	Legube et al., 2004
Phosphatases	PP2A	MEFs	Reid et al., 2013
	Wip1	COS-7	Takekawa et al., 2014
De-acetylases	Sirt1	HEK293T	Langley et al., 2014
	HDAC1/2	PC12	Zhang and Chen, 2007
Deubiquitinases	HAUSP	H1299	Li et al., 2002
	USP10	HCT116, H1299	Yuan et al., 2010
Methyltransferases	Smyd2	H1299	Huang et al., 2006
SUMO ligases	Ubc9	HEK293T	Lin et al., 2004
	PIAS1	Sf-9	Kahyo et al., 2001
	TOPORS	HeLa	Weger et al., 2005
	SUMO-1	U2OS, Saos2	Wade et al., 2010; Rodriguez et al., 2014
Others	HMGA1	HEK293T, H1299	Pierantoni et al., 2014
	Pin1, p21, Bax	HCT116	Mantovani et al., 2007
	Bcl-2, Bcl-xL	H1299, HeLa	Mihara et al., 2003
	Anexin A2, PSF	H1299	Sharathchandra et al., 2012
	BRCA2	H1299	Rajagopalan et al., 2010
	Hsp90	PBMCs	Fukumoto and Kiang, 2011
	BRCA1	MEFs	Xu et al., 2001
	TIAF1	U937	Schultz et al., 2004
	CSA, CSB	CS1AN, HeLa	Latini et al., 2011
	PTTG1	HCT116	Bernal et al., 2002
	HBx	Hep3B	Sato et al., 2011
	ARC	H9c2	Li et al., 2008
	SPARC, Pax6	Astrocytes	Tripathi and Mishra, 2010
	caspase3	WM115	Frank et al., 2011
	ASPP	HEK293T, H1299	Mantovani et al., 2007
	Smad2, Smad3	HEK293T	Cordenonsi et al., 2003
	L2DTL/CDT2, PCNA, CUL4A/DDB1	MEFs, HEK293T	Banks et al., 2006
	Daxx, Axin	H1299, HeLa	Li et al., 2007

Based on these works, induction of apoptosis during PCV2 infection is a complex process that may involve cross-talk between the intrinsic and the extrinsic apoptotic pathway. Certainly, the mechanistic role of PCV2 ORF3 protein in the regulation of apoptosis should be investigated in more detail in future studies.

To date, more than twenty proteins have been shown to be associated with pPirh2 (Jung et al., 2012). Additionally, p53 is a highly connected protein that could form physical complexes with a variety of cellular proteins (Collavin et al., 2010); currently, more than 320 reported interactions with human p53 are included in the APID web interface^1^, including kinases, phosphatases, acetyltransferases, de-acetylases, ubiquitin ligases, and other proteins. Accordingly, future investigation should consider whether there are other factors regulate PCV2-induced apoptosis by participating in the interactions of pPirh2 and ORF3.

### ORF4 and Its Mechanisms of Apoptosis Regulation

Studies indicated that the ORF4 protein is not required for PCV2 replication in mice or in PK-15 cells, while present research showed it plays a vital role in inhibiting apoptosis after PCV2 infection (He et al., 2013). Subsequently, Gao et al. (2014a) constructed two mutants of PCV2 ORF4: M1-PCV2 and M2-PCV2. By comparing the ORF3 mRNA levels of the wild-type and ORF4-deficient viruses in PK-15 cell, it was found that the ORF3 transcription levels of both ORF4 mutants were enhanced, indicating that the ORF4 protein may play an important role in preventing PCV2-induced apoptosis via inhibiting ORF3 transcription. Significant increases in caspase-8 and caspase-3 activities in both ORF4 mutants compared to wild-type PCV2 further confirmed this (Gao et al., 2014a). Subsequently Lv et al. (2016), revealed a mechanism by which ORF4 exerts cytoprotective function by resisting apoptosis in the early stage of PCV2 infection. Lv et al. (2015b) demonstrated the physical interaction between PCV2 ORF4 protein and FHC for the first time, and found that the decreased concentration of FHC can effectively inhibit the accumulation of reactive oxygen species in PCV2-infected cells, thereby inhibiting apoptosis. Recently, Lin et al. (2018) found that ORF4 is a mitochondrial targeting protein that ultimately induces apoptosis via the mitochondrial pathway by interacting with adenine nucleotide translocase 3 (ANT3).

**FIGURE 2:**
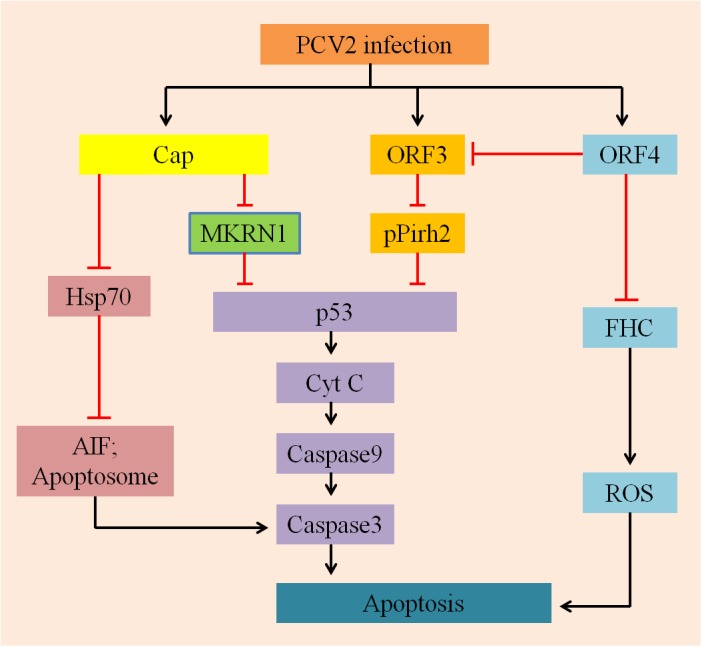
A hypothetical model describes the mechanisms involved in PCV2-ralated proteins induced apoptosis. On the one hand, Cap protein expressed by PCV2, which subsequently inhibits p53 and its downstream pro-apoptotic factors CytC, capases9, and caspase3 via MARNI pathway; it can also suppress Hsp70 and further inhibition the production of AIF and Apoptosome, depressing apoptosis. On the other hand, ORF3 and ORF4 proteins are largely involved in regulating apoptosis induced by PCV2: ORF3 protein interacts with pPirh2 to up-regulate the expression of p53 and its downstream factors to initiate apoptosis; whereas ORF4 protein inhibits apoptosis by suppressing activation of ORF3, it can also interact with FHC to reduce the content of FHC, inhibiting the production of ROS and ultimately suppression apoptosis.

In summary, it is very significant to study how the apoptotic processes are regulated by the proteins of PCV2 to promote its infection (**Figure 2**). In addition to the factors mentioned above, there are other reported mechanisms that could regulate PCV2-induced apoptosis, including different pathways (PERK/eIF2α, PI3K/Akt, and Fas/FasL), regulation of free Ca^2+^ concentration and NF-κB activation. In the following sections, we will briefly review these factors.

## PCV2-Induced Apoptosis Regulated by Different Pathways

### PERK/eIF2α Pathway

**FIGURE 3:**
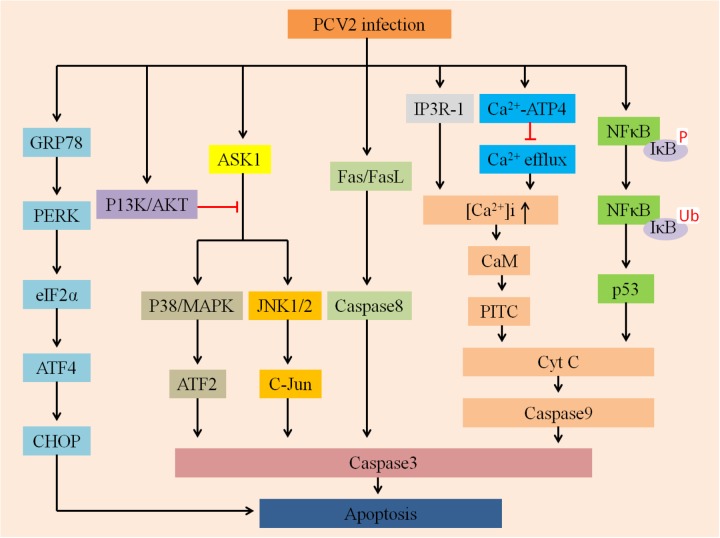
Summarizes multiply host cellular signaling pathways involved in regulating PCV2-induced apoptosis. First, PCV2 infection can activate PERK via PERK-eIF2α-ATF4-CHOP axis and then induce apoptosis, it can further activate JNK/p38 by activating the ASK1 pathway to ultimately promote apoptosis, whereas P13K/AKT plays an opposite role. Second, it can activate Cyt C and caspase-3 via the IP3R-1-Ca^2+^-PITC and NFκB-p53 pathways to activate apoptosis. In addition, PCV2 infection may activate caspase-8 via the Fas/FasL axis of the death receptor pathway to promote apoptosis.

Mounting evidence indicates that a wide variety of viruses could disturb ER homeostasis and lead to ER stress (Li et al., 2015). To cope with this stress, cells evolve a series of adaptive mechanisms called the UPR (Hetz, 2012). ER stress activates three branches of the UPR: PERK (Shen et al., 2005), IRE1 (Chen and Brandizzi, 2013), and ATF6 (Yan et al., 2002). Zhou et al. (2016) demonstrated that PCV2 initiated UPR by activating the PERK/eIF2α pathway instead of IRE1 or ATF6 pathways, ultimately promoting viral replication in PK-15 cells (**Figure 3**). Since PERK/eIF2α further activates downstream factors ATF4 and CHOP, so PCV2 infection can selectively activate apoptosis via the PERK-eIF2α-ATF4-CHOP axis. The findings provide a basis for demonstrating that ER stress of apoptotic responses plays an important role in the pathogenesis of PCV2 infection.

### PI3K/Akt and ASK1 Pathway

The phosphatidylinositol 3-kinase PI3K/Akt pathway plays a vital role in multiple physiological processes, such as inflammation suppression, carbohydrate metabolism, and cellular proliferation (Hsu et al., 2010). The PI3K/Akt pathway is also an indispensable target for a variety of viruses to inhibit apoptosis (Cooray, 2004; Shin et al., 2007; Soares et al., 2009). For example, PRRSV can trigger the PI3K/Akt pathway to augment viral replication and promote cell survival (Wang et al., 2014). Recently, Wei et al. (2012a) found that PCV2 can transiently activate the PI3K/Akt pathway, and the activated PI3K/Akt pathway could suppress premature apoptosis, thereby improving virus growth (**Figure 3**). However, in the early stage of PCV2 infection, inhibition of PI3K activation greatly enhanced apoptotic responses, mainly manifested by the cleavage of caspase3 and poly-ADP ribose polymerase as well as DNA fragmentation. The ASK1 plays a target role in the induction of apoptosis as an upstream enzyme that activates the p38/MARK and JNK pathways (Gan et al., 2016). During PCV2 infection, PI3K was activated first, followed by phosphorylation of Akt. Activated Akt inhibits the production of pro-apoptotic proteins such as JNK and p38/MAPK, thereby suppressing JNK- and p38-dependent apoptosis (Wei et al., 2013).

Interestingly, a previous study demonstrated that PCV2 infection regulates apoptosis by activating the p38/MAPK and JNK1/2 cellular stress pathways (Tibbles and Woodgett, 1999; Kyriakis and Avruch, 2001; Wada and Penninger, 2004; Wei et al., 2009). In the absence of stress, non-phosphorylated JNK bonds to p53, resulting in ubiquitination of p53 followed by proteasomal degradation (Fuchs et al., 1998a,b). In contrast, dissociation of p53 can be mediated by phosphorylated JNK, thus promoting p53 stabilization (Fuchs et al., 1998b). Additionally, p38/MAPK kinase plays a role not only in phosphorylation of p53 but also in transcription of p53-regulated Bax (Bulavin et al., 1999; Huang et al., 1999). Taken together, the activation and phosphorylation of p38/MARK and JNK after PCV2 infection might contribute to p53 stabilization, finally leading to apoptosis (Wei et al., 2009).

### Fas/FasL Pathway

Chang et al. (2007a) evaluated and compared the effects of infection of both PCV2 and PRRSV, individually or together, on co-cultured splenic (SLs), peripheral blood (PBLs) lymphocytes and swine splenic macrophages (SMs) *in vitro*. The expression levels of Fas ligant (FasL) and Fas were significantly increased after PRRSV alone- and PCV2 and PRRSV dually inoculated groups, and the latter was more obvious, while increased Fas/FasL futher mediated apoptosis. Fas is also termed as CD95 (APO-1) and is one of the death receptors, these receptors include TNF-R1, CD95 (APO-1/Fas), DR3 (APO-3/TRAMP/Wsl-1/LARD), DR4 (TRAIL-R1), and so on. Han et al. (2010) confirmed that Fas could trigger apoptosis by binding to its cognate ligand, FasL. Thus, PCV2 infection may be associated with Fas/FasL-mediated apoptosis (**Figure 3**). However, the hypothesis of the mechanism is still poorly understood and need to be further demonstrated.

### NF-κB Pathway

The transcription factor NF-κB is commonly activated during viral infection and is a key molecule that regulates a variety of cellular signal transduction pathways (Bonizzi and Karin, 2004; Hayden and Ghosh, 2004). For example, Dengue virus, Reovirus, infectious bursal disease virus, Hepatitis B virus and Sindbis virus have been confirmed to trigger apoptosis via activating NF-κB (Lin et al., 1998; Connolly et al., 2000; Jan et al., 2000; Liu and Vakharia, 2006; Pan et al., 2011; Chen et al., 2013). In these processes, NF-κB serves as a pro-apoptotic factor which is able to activate the p53 signaling pathway (Fujioka et al., 2004).

The present study found that after PCV2 infected cells, NF-κB was activated simultaneously with viral replication, which was characterized by translocation of NF-κB from the cytoplasm to the nucleus, degradation and phosphorylation of IκBα protein and increased DNA binding activity. However, treatment of cells with CAPE, a selective inhibitor of NF-κB activation, reduced progeny production and virus protein expression followed by decreasing caspase activity, indicating the importance of NF-κB in inducing apoptosis (Wei et al., 2008). However, the exact details still to be further demonstrated. According to the above discussions, there are many factors and multiply pathways participate in regulating apoptosis induced by PCV2 (**Figure 3**).

## PCV2-Induced Apoptosis Regulated by Calcium

Calcium ions (Ca^2+^) are participated in multiple cellular physiological processes, such as cytoplasmic Ca^2+^ signaling, ATP production, hormone metabolism and apoptosis induction (Drago et al., 2011). The intracellular free Ca^2+^ ([Ca^2+^]i) can activate apoptosis by regulating numerous calcium-sensitive enzymes and can also activate the mitochondrial apoptotic pathway via its accumulation in the mitochondria (Hajnoczky et al., 2003; Pathak et al., 2013). Lv et al. (2012) found that PCV2 could lead to apoptosis of lymphocytes, this apoptotic mechanism is affected by the increased [Ca^2+^]i and is associated with the calmodulin (Lee et al., 2009) protein. Possible mechanisms of [Ca^2+^]i induction include the suppression of Ca^2+^ efflux by regulation of the Ca^2+^-ATPase transporter on cytomembranes, and/or the induction of Ca^2+^ influx by promoting Ca^2+^ release from the ER by increased expression of IP3R (Lv et al., 2012). IP3R can regulate the mobilization of Ca^2+^ (Berridge, 2005), Ca^2+^ released from the ER could activate the PTPC on mitochondria, causing Cyt c release and inducing apoptosis (**Figure 3**; Garrido et al., 2006).

## Conclusion

Apoptosis is a very important host defense mechanism that contributes to remove infected, damaged and excess amounts of cells. The virus must evade host defense mechanisms to proliferate and spread. Infection with PCV2 has been demonstrated to trigger several signaling pathways such as PERK/eIF2α and PI3K/Akt pathway (Wei et al., 2012a; Zhou et al., 2016), resulting in activation or suppression of apoptosis. On the other hand, to cope with the apoptotic responses caused by viral infections, many viral proteins interact with apoptotic signals molecules to regulate apoptosis. There may be a discrepancy between induction and inhibition of apoptosis after PCV2 infection, as the experimental situation can be different, and close relationships between apoptosis and other factors that regulate cell fate, such as Ca^2+^, can make it more complicated and difficult.

**FIGURE 4:**
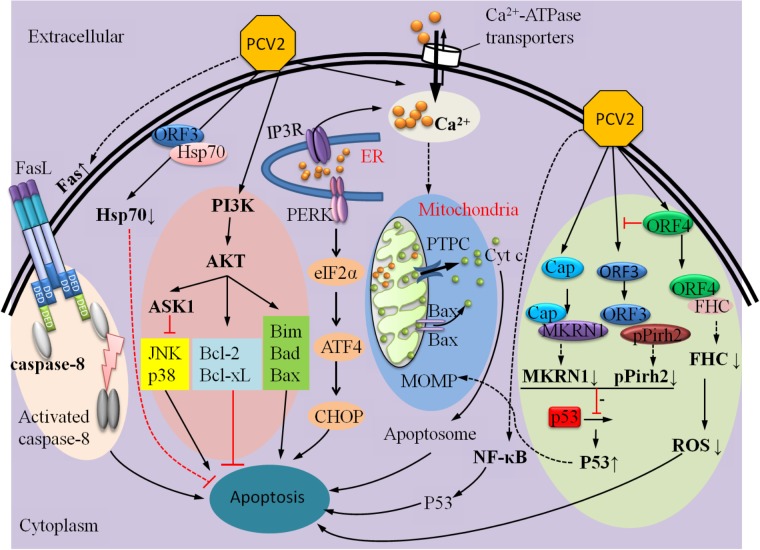
A summary of mechanisms that can regulate PCV2-induced apoptosis.

This review is the first glimpse of PCV2 infection-induced apoptosis based on a wide array of reported works concerning PCV2 infection. It summarized currently findings which are involved in PCV2 infection-induced apoptosis, containing a vast panel of distinct pro-apoptotic and anti-apoptotic mechanisms (**Figure 4**). In the future, more attention should be taken on host-virus interaction. Further investigation that effect of different isoforms of PCV2, PCV1, and PCV3 on PCV-induced apoptosis should be done. Taking the above ideas into consideration will help us reach a deeper understanding of the molecular mechanisms of PCV2-induced apoptosis and open a new gate for further studies on the pathogenesis of PCV2.

## Author Contributions

PL and RJ contributed ideas for the review. YP wrote the manuscript and produced the figures. RJ, MW, ZY, and AC edited and revised the manuscript.

## Conflict of Interest Statement

The authors declare that the research was conducted in the absence of any commercial or financial relationships that could be construed as a potential conflict of interest.

## Footnotes

1 http://bioinfow.dep.usal.es/apid/index.html

## Abbreviations

AIF: apoptosis-inducing factor
Apaf-1: apoptosis-protease activating factor-1
ASK1: apoptosis signal-regulating kinase 1
ATF6: activating transcription factor 6
[Ca^2+^]i: the intracellular free Ca^2+^ concentration
Cap: capsid
Cyt c: cytochrome c
ER: endoplasmic reticulum
FHC: ferritin heavy chain
gC1qR: globular heads of complement component C1q
Hsp40: heat-shock protein 40
Hsp70: heat-shock protein 70
IP3R: inositol 1,4,5-trisphosphate receptor
IRE1: inositol requiring enzyme 1
JNK: c-Jun NH2-terminal kinase
MDM2: murine double minute 2 gene
MKRN1: makorin-1 RING zinc-finger protein
MOMP: mitochondrial outer membrane permeabilization
NAP1: nucleosome assembly protein-1
NPM1: nucleophosmin-1
ORFs: open reading frames
p38/MARK: p38 mitogen-activated protein kinase
Par-4: prostate apoptosis response-4 protein
PAMs: pulmonary alveolar macrophages
PCV2: Porcine Circovirus type 2
PCVAD/PCVD: porcine circovirus-associated diseases
PERK: PKR-like ER kinase
PMWS: post-weaning multi systemic wasting syndrome
pPirh2: porcine Pirh2
PTPC: permeability transition pore complex
Rep: replicase
RGS: regulator of G protein signaling
UPR: unfolded protein response
WNV: West Nile virus.
